# Supplementary material for: Ancient dolphin genomes reveal rapid repeated adaptation to coastal waters
Source: Nat Commun. 2023 Jul 18;14:4020. doi: 10.1038/s41467-023-39532-z (PMC10354069; doi:10.1038/s41467-023-39532-z)
Supplement: Supplementary file 1 — Supplementary Information [file 41467_2023_39532_MOESM1_ESM.pdf]

**Ancient dolphin genome reveals rapid repeated adaptation to coastal waters**

**Supplementary information**

Marie Louis[1,2,3,4]*, Petra Korlević[5,6]*, Milaja Nykänen[7,8]*, Frederick Archer[9], Simon Berrow [10,11], Andrew Brownlow[12], Eline D. Lorenzen[2], Joanne O'Brien [10,11], Klaas Post[13], Fernando Racimo[2], Emer Rogan[8], Patricia E. Rosel[14], Mikkel-Holger S. Sinding[15], Henry van der Es[13], Nathan Wales[16], Michael C. Fontaine[3,17]**, Oscar Gaggiotti[1]**, Andrew D. Foote[18,19]**

**These authors contributed equally*

***These authors jointly supervised this work*

1. Scottish Oceans Institute, University of St Andrews, East Sands, St Andrews KY16 8LB, Scotland, UK
2. Globe Institute, University of Copenhagen, Øster Voldgade 5, 1350 Copenhagen, Denmark
3. Groningen Institute for Evolutionary Life Sciences (GELIFES), University of Groningen, PO Box 11103 CC, Groningen, The Netherlands
4. Greenland Institute of Natural Resources, Kivioq 2, Nuuk 3900, Greenland
5. Max Planck Institute for Evolutionary Anthropology, Deutscher Platz 6, 04103 Leipzig, Germany
6. Wellcome Sanger Institute, Wellcome Genome Campus, Hinxton, Cambridge, CB10 1SA, UK
7. Department of Environmental and Biological Sciences, PO Box 111, FI-80101 Joensuu, Finland
8. School of Biological, Earth and Environmental Sciences, University College Cork, North Mall, Cork, Ireland
9. Marine Mammal and Turtle Division, Southwest Fisheries Science Center, NOAA, 8901 La Jolla Shores Drive, La Jolla, CA 92037, USA
10. Irish Whale and Dolphin Group, Kilrush, Co Clare, Ireland
11. Marine and Freshwater Research Centre, Department of Natural Sciences, School of Science and Computing, Galway-Mayo Institute of Technology, Dublin Road, H91 T8NW Galway, Ireland.
12. Scottish Marine Animal Stranding Scheme, Institute of Biodiversity, Animal Health & Comparative MedicineCollege of Medical, Veterinary & Life Sciences, University of Glasgow
13. Natural History Museum Rotterdam, Westzeedijk 345, 3015 AA Rotterdam, Netherlands
14. Marine Mammal and Turtle Division, Southeast Fisheries Science Center, NOAA, Lafayette, LA 70506, USA
15. Department of Biology, University of Copenhagen, Ole Maaløes Vej 5, 2200 Copenhagen, Denmark.
16. University of York, BioArCh, Environment Building, Wentworth Way, Heslington, York, YO10 5DD
17. MIVEGEC (Université de Montpellier, CNRS 5290, IRD 229) Institut de Recherche pour le Développement (IRD), F-34394, Montpellier, France
18. Molecular Ecology Fisheries Genetics Lab, School of Biological Sciences, Bangor University, Bangor, UK
19. Department of Natural History, Norwegian University of Science and Technology (NTNU), NO-7491 Trondheim, Norway

Corresponding authors: Marie Louis (marielouis17@hotmail.com), Andrew D. Foote (andrew.foote@ntnu.no)

# Supplementary notes

## Mapping statistics

### *Mitogenomes*

The *T. truncatus* mitogenome sequence has 16,388 sites. We called three nucleotides in NMR10151 and one nucleotide in NMR2273 as Ns as there was not a single nucleotide representing > 75% of the reads. There were no ambiguities in SP1060 and NMR10326. In the 60 contemporary samples, there was one ambiguous base (i.e. without a single nucleotide representing > 75% of the reads) in four of the samples (160321, IR33, S37, S41), which was changed to N. This could represent heteroplasmy.

Coverage of the mitogenomes for the ancient samples was as follows: SP1060: 160.4x, NMR2273: 9.9x, NMR10326: 27.6x and NMR10151: 2.3x.

Average mitochondrial genome coverage of the 57 contemporary samples from Louis *et al.* (2021) [1] was 1446.2x (SD=595.1) and the three new contemporary samples from this study was 109.2x (SD=14.8).

Proportion of missing bases (N) was 0.0001 in the 57 contemporary samples (122 bases over all samples), 0.006 in the three new contemporary genomes (3 bases overall all samples), 0.0002 for SP1060 (4 bases), 0.003 for NMR10326 (50 bases), 0.006 for NMR2273 (100 bases) and 0.25 for NMR10151 (4,264 bases).

### *Nuclear results statistics*

For SP1060, endogenous content was 28% when mapping to the bottlenose dolphin reference genome and 24.1% when mapping to the killer whale reference genome, and coverage was 3x (Supplementary Table 2). Mapping statistics for all samples are detailed in Supplementary Table 2.

## Mitochondrial DNA phylogeny

### *Delphinid phylogeny*

The average overall rate for substitutions per site per million years (clock rate) for the Delphinids in both phylogenies (with and without the sample 117699) was estimated as 6.670

$\times 10^{-3}$ (95% HPDI: $4.877 \times 10^{-3} - 8.835 \times 10^{-3}$), and the TMRCA of the two most divergent *T. truncatus* samples was estimated as 0.916 Mya (95% HPDI: 0.630 – 1.234 Mya) for the phylogeny without 117696 and as 1.174 Mya (95% HPDI: 0.814 – 1.567 Mya) for the tree including 117696. The Effective Sample Size (ESS) values for the different parameters in the combined runs were all >250, with most of them >3,000, indicating no autocorrelation between samples.

*T. truncatus phylogeny*

The ESS values for the model parameters in the *T. truncatus* tree models were all >1500 (in individual runs), indicating no sign of autocorrelation between samples and a good convergence of chains. The scenario with the highest posterior probabilities of deeper (older) nodes included all of the subfossil samples and the contemporary sample 117696. We will therefore present only the results from this model, however, it is important to note that all model scenarios (all codon and 3rd codon) placed the coastal NWA samples into a separate monophyletic clade, concordant with recent studies [1,2]. In addition, the placement of the subfossil samples was identical in all model scenarios (Supplementary Figure 2).

The average clock rate for the all-codon bottlenose dolphin phylogenetic model was estimated as $7.026 \times 10^{-3}$ substitutions/site/Myr with 95% HPDI of $4.579 \times 10^{-3} - 9.949 \times 10^{-3}$. The summary consensus tree consisting of *T. truncatus* samples indicates that the coastal WNA clade has the oldest coalescence time of 0.8 My (95% HPDI: 0.493 – 1.100 My) (Supplementary Figure 2). A clade consisting of samples collected from the eastern North Pacific, however, has a slightly younger mean coalescence time of 0.704 My (95% HPDI: 0.442 – 0.984 My). All of the dated subfossil samples are placed in a clade that include mostly contemporary samples collected from pelagic North Atlantic, however, there are also three samples in this clade originating from the Mediterranean and Black Seas (Supplementary Figure 2). As in a previous study [3], incomplete lineage sorting is evident in the coastal eastern North Atlantic sequences.

## Population structure

*Projection of the ancient samples on the Principal Components (PC)*

The position of SP1060 is consistently intermediate between the pelagic populations and the two North Atlantic coastal populations in all analyses on the first and second PC, that is when using called diploid genotypes (Supplementary Figure 4), or pseudo-haploid calls (Figure 1C, Supplementary Figure 4), removing (Figure 1C, Supplementary Figure 6) or keeping transitions (Supplementary Figure 4). The coastal and the pelagic samples separate on PC3, with the ancient sample being intermediate (Supplementary Figure 5).

While the position of SP1060 remains relatively unchanged when mapping to the bottlenose dolphin (Supplementary Figure 6) or the killer whale (Figure 1c) reference genomes, we note some reference bias when mapping the ultra-low coverage samples to the bottlenose dolphin reference genome (Supplementary Figure 6). The position of the three samples is shifted towards being closer to the WNAc individuals, the reference genome being from a WNAc population, than when mapping to the killer whale reference genome (Figure 1c), in particular for the lowest coverage sample NMR10151.

*Factorial analysis (tfa)*

In the tfa analysis, factor 1 separates the Pacific and the Atlantic population, while factor 2 separates the coastal populations from the ENA and WNA. The tfa analysis also indicates that SP1060 is intermediate between the two Atlantic coastal populations and the pelagic populations (Supplementary Figure 7).

*ANGSD single-read sampling PCA approach*

Using the single read sampling method in ANGSD we got relatively similar PCA results than with smartpca. The position of SP1060 as intermediate between the two Atlantic coastal populations and the pelagic is unchanged (Supplementary Figure 8). We find that the two younger samples (SP1060 and NMR10151) are closer to the coastal populations than the two older samples (NMR2273 and NMR10326, Supplementary Figure 8).

## Evolutionary relationships

### *D-statistics analysis*

The *D*-statistics results were consistent for the data mapped to the bottlenose dolphin reference genome (Supplementary Figure 9) and the killer whale reference genome (Supplementary Figure 10), with the transitions (Supplementary Figures 9a and 10a) and removing the transitions (Supplementary Figures 9b and 10b). We note some slight reference bias towards the WNAc when mapping to the WNAc bottlenose reference genome, in particular when including the transitions. The value of the statistic *D*(ENAc,WNAc; SP1060,*orca*) is significantly negative, indicating that SP1060 is more closely related to the ENAc dolphins than the WNAc dolphins. This pattern is the strongest when mapping to the killer whale genome, with and without transitions (Supplementary Figure 10), and the lowest when mapping to the bottlenose dolphin reference and when keeping the transitions (Supplementary Figure 9b), highlighting the need to take reference bias and damage patterns into account. Similarly, ENAc shared a higher excess of derived alleles with SP1060 than WNAc, with the value of statistics of the form *D*(coastal,pelagic; SP1060,*orca*) being lower when the eastern Atlantic populations are included than when the western are. This pattern is the strongest when mapping to the killer whale reference genome.

### *Admixture graph analyses*

We find similar graph topologies when using the pseudohaploid data mapped to the killer whale genome and including both SP1060 and the contemporary Atlantic populations (Figure 2) and when using called genotypes and the contemporary Atlantic populations only (Supplementary Figure 11).

## Inferences on the SNPs under parallel linked selection

### *Heterozygosity estimation*

Heterozygosity (He) was significantly higher in coastal than pelagic population in the ENP ($t = 6.54$, $df = 12.07$, $P < 0.01$), in the ENA ($W = 130$, $P < 0.01$) and WNA ($t = 22.23$, $df = 10.01$, $P < 0.01$).

## Supplementary Figures

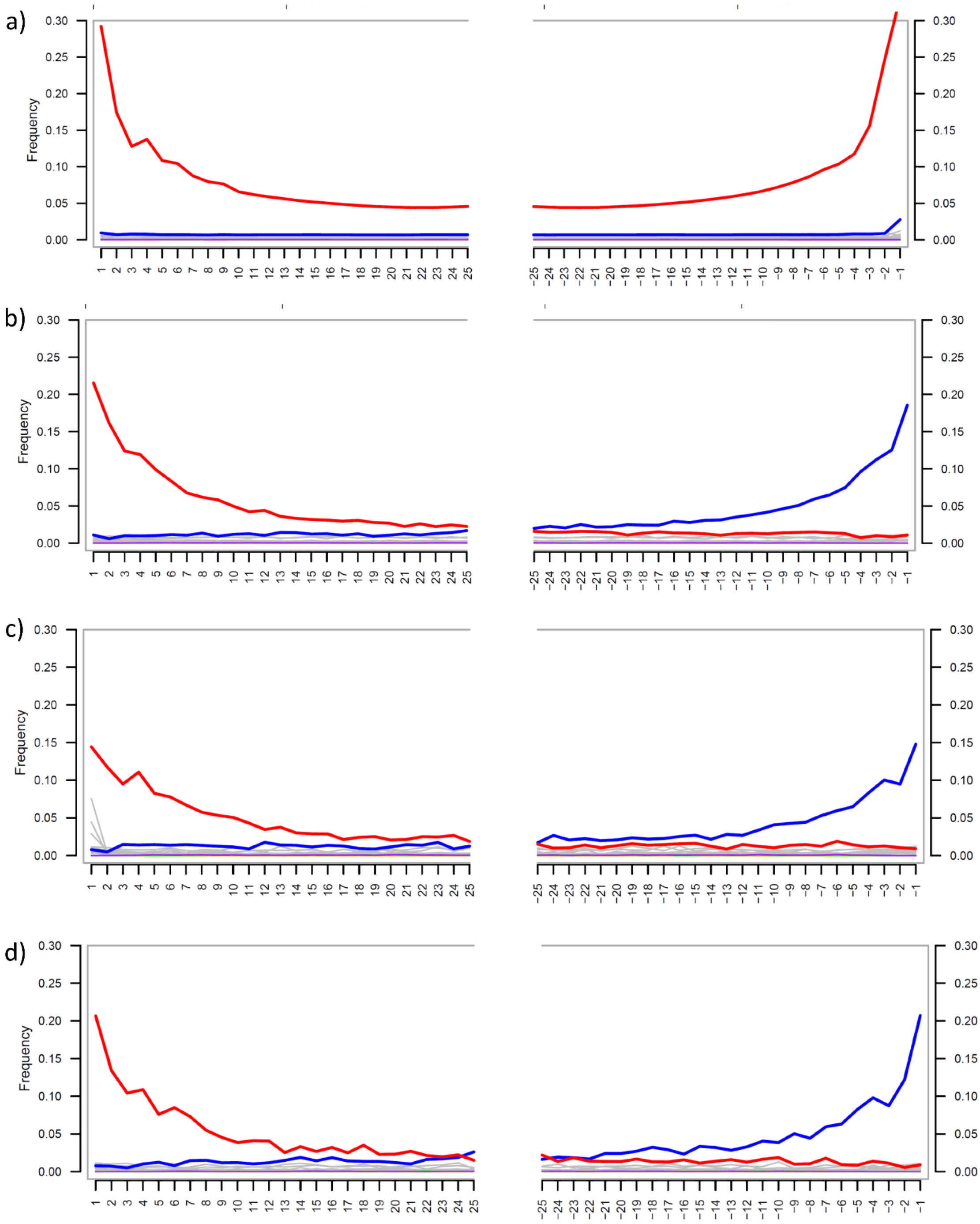


Supplementary Figure 1. Misincorporation pattern plot obtained with MapDamage for the first and last 25 bases for a) SP1060, b) NMR10326, c) NMR2273 and d) NMR10151. It shows the percentage of sites containing a nucleotide change from the killer whale reference sequence along the DNA fragment with: red indicating C to T transitions, blue indicating G to A transitions. SP1060 doesn't show the complementary G->A excess due to being a single stranded library. Grey represents all other substitutions and purple insertions relative to the reference. Source data are provided in the DataSuds repository and NCBI.

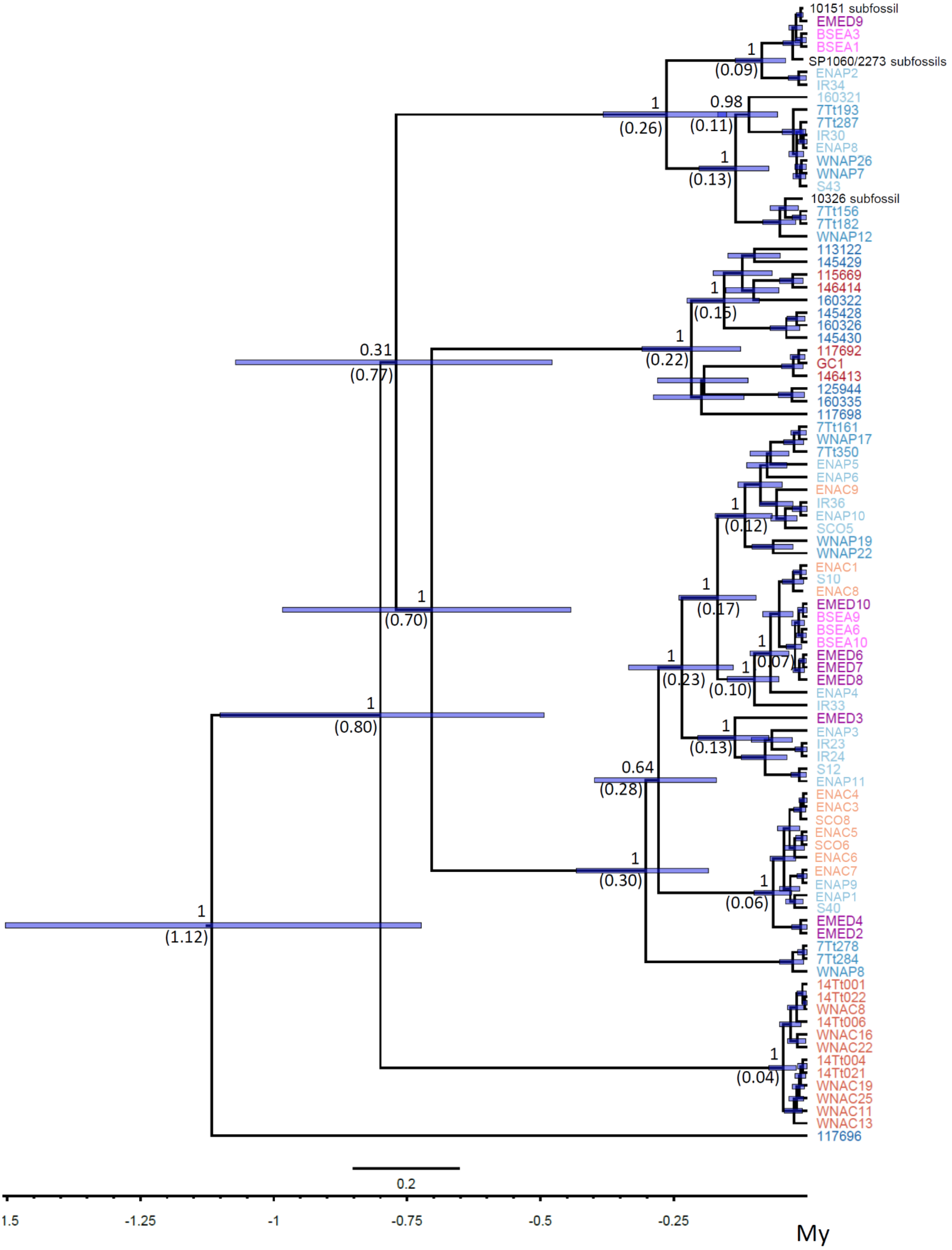


Supplementary Figure 2. Time-calibrated phylogenetic tree for the *Tursiops truncatus* samples (haplotypes) estimated with BEAST2 coalescent model with constant populations and using 13 mitochondrial protein coding genes. The numbers above and below nodes represent the node posterior probability and mean node age (in brackets), respectively, and the bars depict 95% HPDI in node TMRCA. The ancient subfossil samples are highlighted in

black colour. The pelagic populations are coloured in different shades of blue, and the coastal populations of red. Note that the eastern Mediterranean Sea samples are in purple and the Black Sea in pink. Individuals from the eastern Mediterranean Sea and Black Sea are believed to be coastal [4]. New mitochondrial genome haplotypes have been assembled from data from Louis *et al.* (2021) [1] and we also used publicly available mitochondrial genome haplotypes from Nykänen *et al.* (2019) [3] and Moura *et al.* (2013) [4]. Source data are provided in the DataSuds repository.

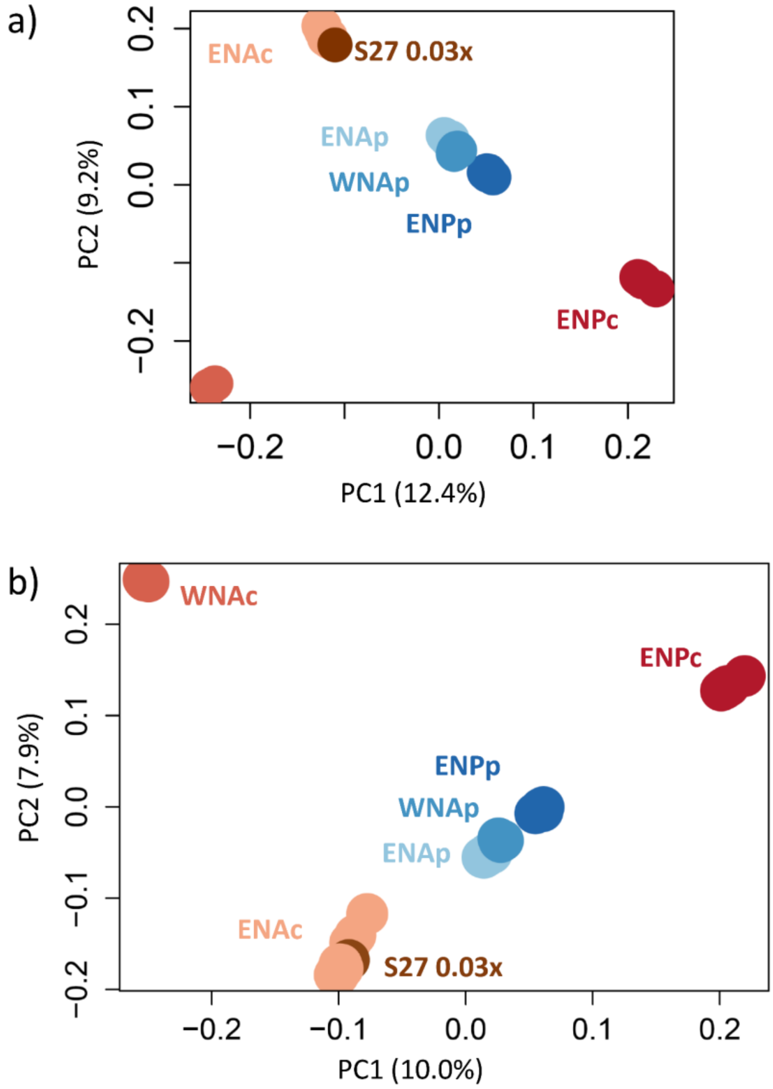


Supplementary Figure 3. a) Principal component analysis of pseudo-haploid data from 55 contemporary and 1 projected downsampled sample to 0.03x (S27 from the ENAc), mapped to the killer whale reference genome, showing first and second principal components (PCs) based on 612,694 SNPs. Populations include coastal and pelagic ecotypes from the eastern

North Atlantic (ENAc and ENAp), western North Atlantic (WNAc and WNAp) and eastern North Pacific (ENPc and ENPp). b) Principal component analysis using the single read sampling in ANGSD showing the first and second components for S27 (ENAc) downsampled to 0.03x, 59 contemporary samples mapped to the killer whale reference genome with relaxed parameters, removing transitions, for a total of 2,463,039 SNPs. Source data are provided in the Source Data file.

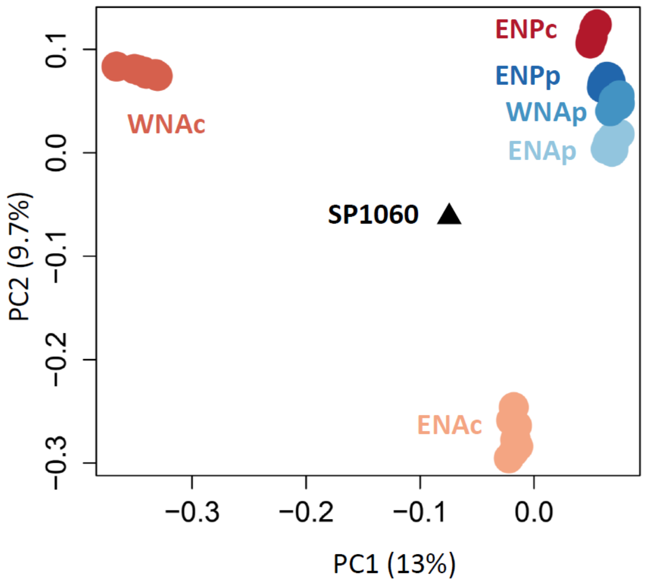


Supplementary Figure 4. Principal component analysis (PCA) projections of SP1060 (black triangle), mapped to the bottlenose dolphin reference genome using BWA relaxed parameters for ancient DNA, on the principal components segregating the contemporary genome with smartpca using call genotypes and including transversions and no missing data in SP1060 for a total of 112,706 SNPs. First and second components are shown. Populations include coastal and pelagic ecotypes from the eastern North Atlantic (ENAc and ENAp), western North Atlantic (WNAc and WNAp) and eastern North Pacific (ENPc and ENPp). Source data are provided in the Source Data file.

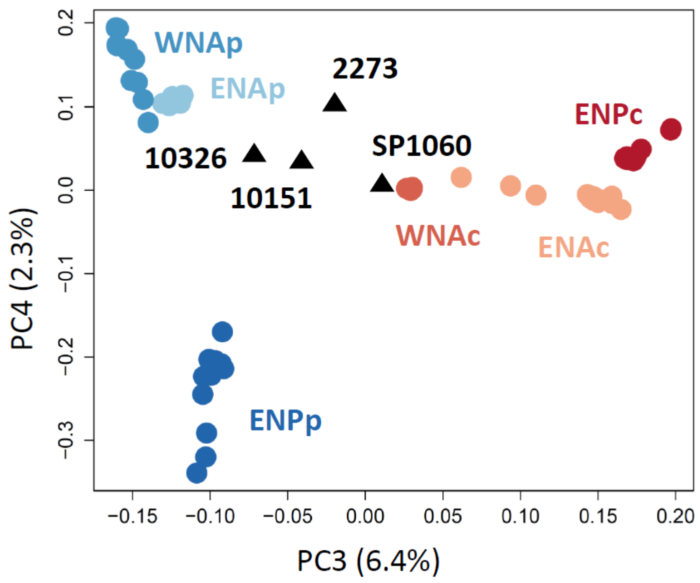


Supplementary Figure 5. Principal component analysis (PCA) projections of the four ancient samples (black triangles), mapped to the killer whale reference genome, on the principal components segregating the contemporary genomes using pseudo-haploid data and removing transversions for a total of 624,969 SNPs. Third and fourth components are shown. Populations include coastal and pelagic ecotypes from the eastern North Atlantic (ENAc and ENAp), western North Atlantic (WNAc and WNAp) and eastern North Pacific (ENPc and ENPp). Source data are provided in the Source Data file.

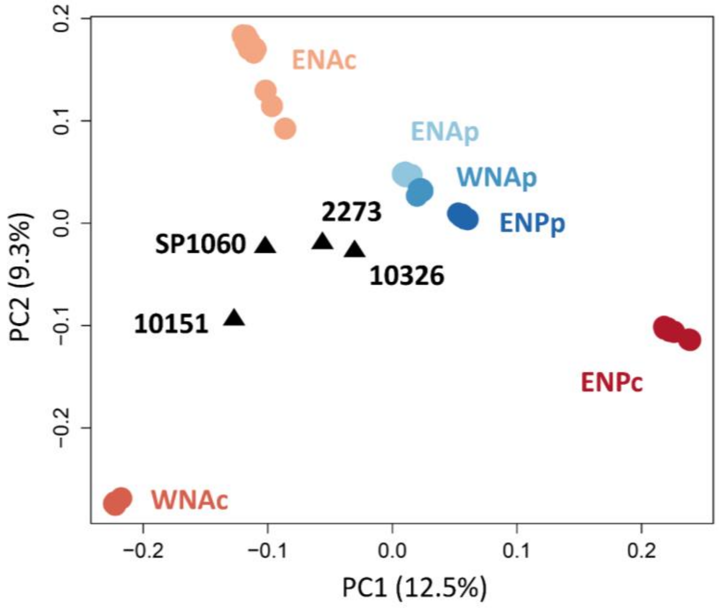


Supplementary Figure 6. Principal component analysis (PCA) projections of the four ancient samples (black triangles), mapped to the bottlenose dolphin reference genome using BWA

relaxed parameters for ancient DNA, on the principal components segregating the contemporary genomes using pseudo-haploid data and removing transversions for a total of 885,285 SNPs. First and second components are shown. Populations include coastal and pelagic ecotypes from the eastern North Atlantic (ENAc and ENAp), western North Atlantic (WNAc and WNAp) and eastern North Pacific (ENPc and ENPp). Source data are provided in the Source Data file.

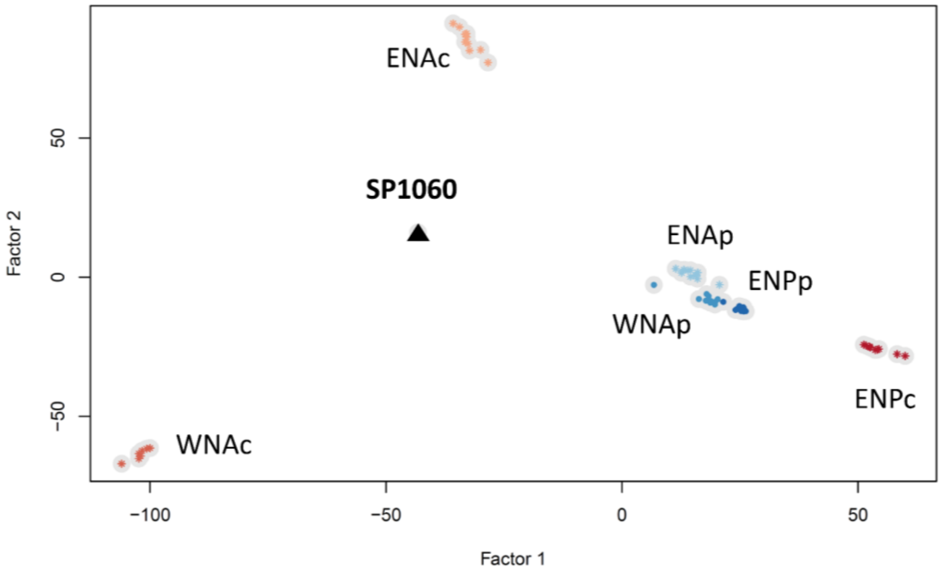


Supplementary Figure 7. Factor analysis of 57 contemporary common bottlenose dolphins and one ancient common bottlenose dolphin of age 5,979-5,626 years BP. 112,506 SNPs with no missing data in SP1060 (black triangle) were included, missing genotypes were imputed in the contemporary individuals. Data was mapped to the bottlenose dolphin reference genome with relaxed parameters. Populations include coastal and pelagic ecotypes from the eastern North Atlantic (ENAc and ENAp), western North Atlantic (WNAc and WNAp) and eastern North Pacific (ENPc and ENPp). Source data are provided in the DataSuds repository.

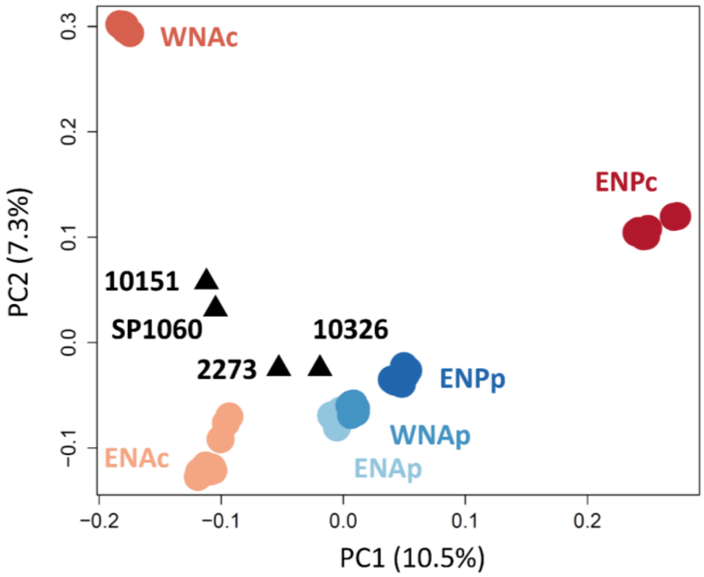


Supplementary Figure 8. Principal components analyses using the single read sampling in ANGSD showing the first and second components for the 60 contemporary samples, SP1060, NMR2273, NMR10326 and NMR10151 (ancient samples are indicated with black triangles) mapped to the bottlenose dolphin reference genome with relaxed parameters, removing transitions, for a total of 16,286 SNPs. Populations include coastal and pelagic ecotypes from the eastern North Atlantic (ENAc and ENAp), western North Atlantic (WNAc and WNAp) and eastern North Pacific (ENPc and ENPp). Source data are provided in the Source Data file.

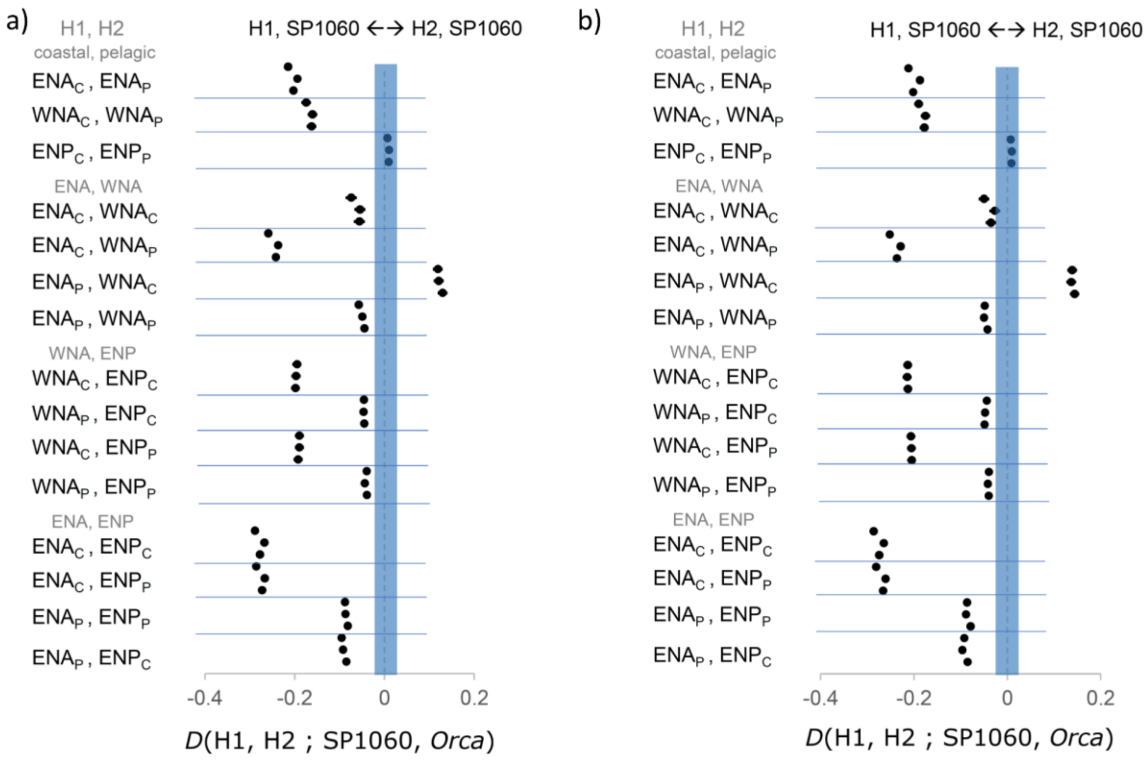


Supplementary Figure 9. Plots of the *D*-statistic (ABBA-BABA) test *D*(H1,H2; SP1060, *Orca*) for the dataset mapped to the bottlenose dolphin reference genome with relaxed parameters a) without the transitions and b) with the transitions. All possible 15 combinations of two contemporary dolphin populations were included as the in-group (H1 and H2) and the killer whale "*Orca*" as the outgroup. For each combination of in-groups, three comparisons with different individuals were computed. Blue shading indicates non-significant results, $-3 > Z < 3$. SP1060 did not share any significant excess of derived alleles (non-significant *D* of 0.010-0.12) with either the ENPc and ENPp, further confirming independent evolution of coastal populations in the Atlantic and the Pacific (see Louis *et al.* (2021) [1]). Populations include coastal and pelagic ecotypes from the eastern North Atlantic (ENAc and ENAp), western North Atlantic (WNAc and WNAp) and eastern North Pacific (ENPc and ENPp). Source data are provided in the Source Data file.

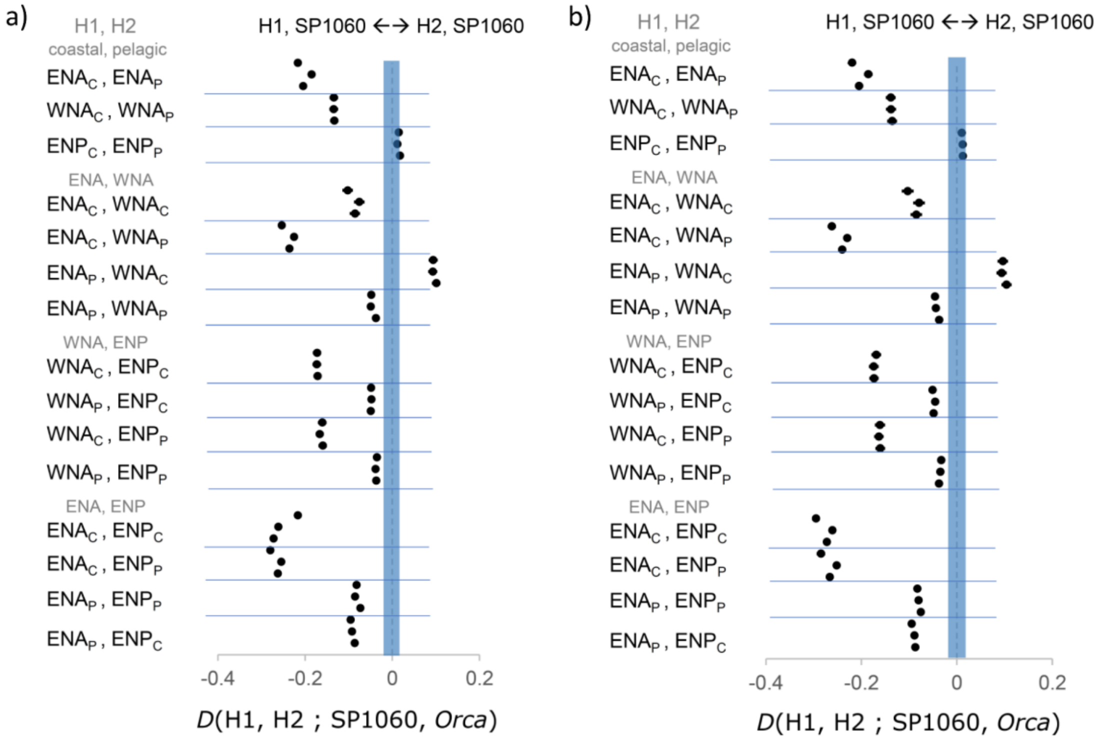


Supplementary Figure 10. Plots of the *D*-statistic (ABBA-BABA) test *D*(H1,H2; SP1060, *Orca*) for the dataset mapped to the killer whale reference genome a) without the transitions and b) with the transitions. All possible 15 combinations of two contemporary dolphin populations were included as the in-group (H1 and H2) and the killer whale "*Orca*" as the outgroup. For each combination of in-groups, three comparisons with different individuals were computed. Blue shading indicates non-significant results, $-3 > Z < 3$. Populations include coastal and pelagic ecotypes from the eastern North Atlantic (ENAc and ENAp), western North Atlantic (WNAc and WNAp) and eastern North Pacific (ENPc and ENPp). Source data are provided in the Source Data file.

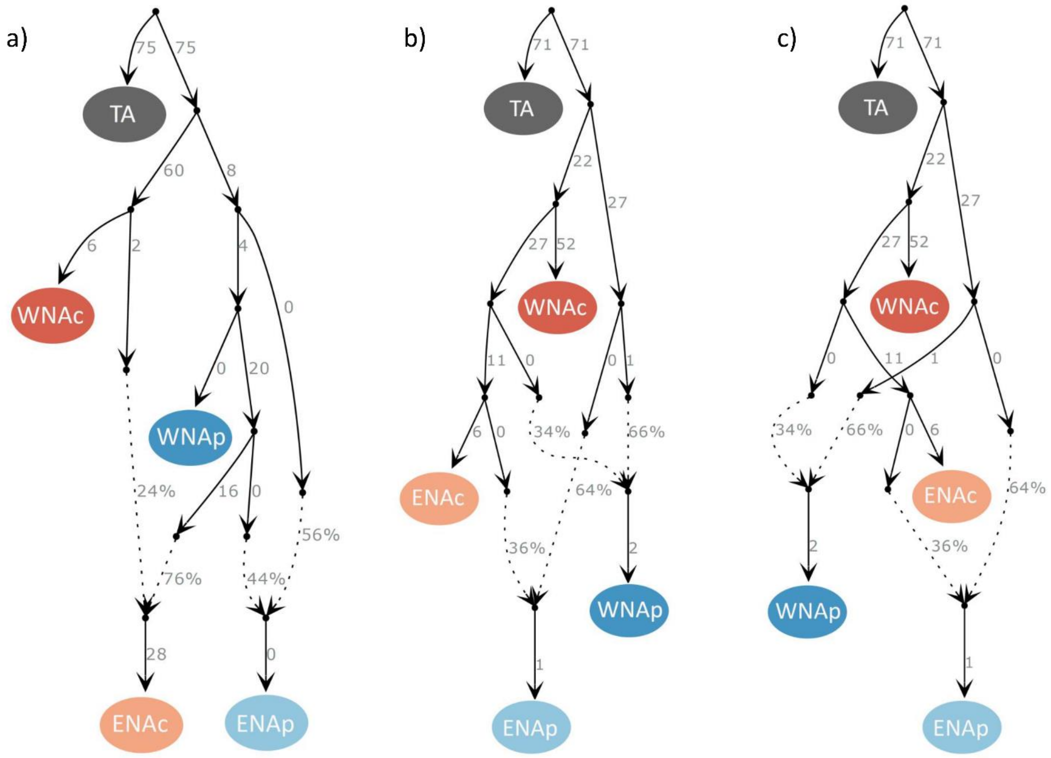


Supplementary Figure 11. Evolutionary relationships between the North Atlantic contemporary bottlenose dolphin populations. Populations include eastern North Atlantic coastal (ENAc) and pelagic (ENAp) populations, and western North Atlantic coastal (WNAc) and pelagic (WNAp) populations, and the outgroup is the Indo-Pacific bottlenose dolphin (*Tursiops Aduncus*, TA). Admixture graphs were built using called genotypes with data mapped to the bottlenose dolphin reference genome including 213,488 SNPs. The graphs a), b) and c) were the three graphs out of all possible graph combinations presenting no outlier *f*-statistics (i.e. all |Z| were <3). Continuous lines indicate phylogenetic relationships between populations/samples and the numbers at their right side the estimated genetic drift. Dotted lines show admixture edges and the number at their right side the percentage of ancestry deriving from each lineage. The Bayes Factors showed a non-significant support for the first graph (a) in comparison to the other “mirror” two (b and c), which have similar log likelihoods (Bayes Factor of 0.88). Source data are provided in the DataSuds repository.

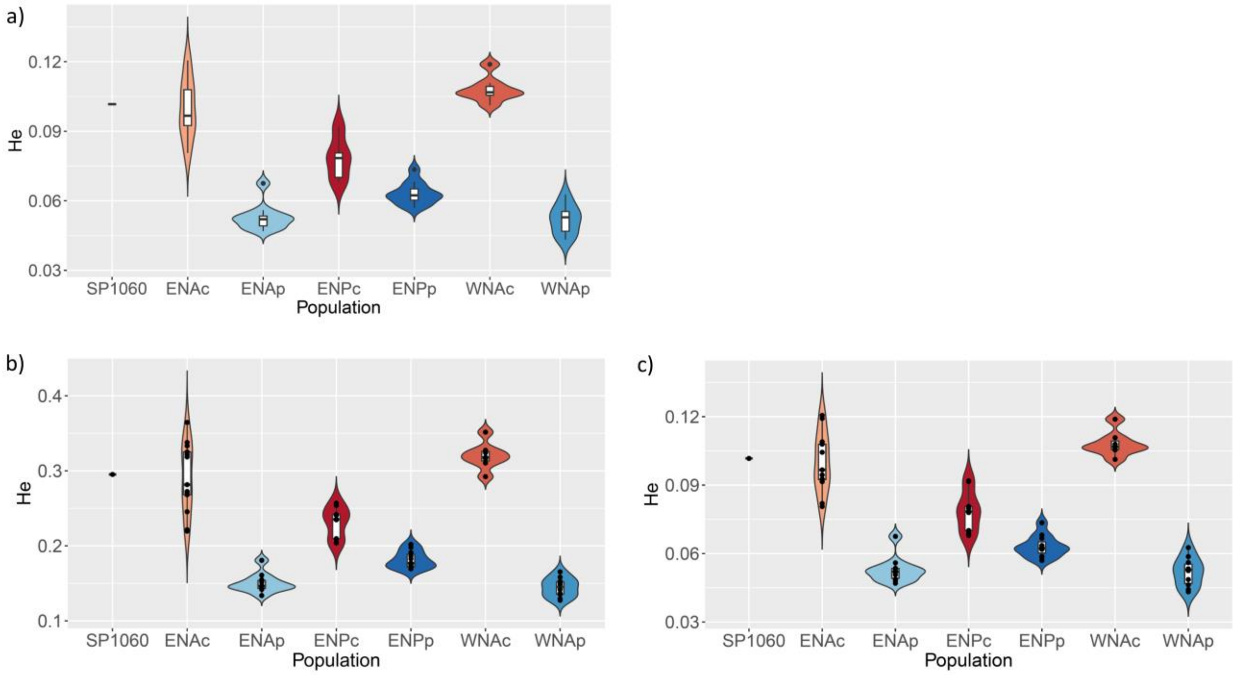


Supplementary Figure 12. a) Heterozygosity (He) estimated for the sites under parallel linked selection for each population removing the transitions; the violin plots indicate the kernel probability density of the data, the box indicates the interquartile range, and the horizontal marker indicates the median of the data. Populations include coastal and pelagic ecotypes from the eastern North Atlantic (ENAc and ENAp), western North Atlantic (WNAc and WNAp) and eastern North Pacific (ENPc and ENPp). Note that we have only one data point for the ancient individual SP1060. Heterozygosity is significantly higher in coastal than pelagic populations as shown by two-sided t-tests or wilcoxon tests in the ENP (ENPc n= 9, ENPp n= 11, t = 4.67, df = 11.71, P = 0.0006), ENA (ENAc n= 13, ENAp n= 10, W = 130, P = 1.75e-06) and WNA (WNAc n= 7, WNAp n= 10, t = 13.41, df = 14.08, P = 1.46e-11). He was estimated by computing individual site-frequency-spectrum using ANGSD v.0.921. SP1060 shows a mean of 0.100 which is very close to the mean of 0.101 for ENAc. b) Heterozygosity (He) estimated for all the sites under parallel linked selection for each population, the figure is the same as Figure 3c, but with all the data points shown. c) Heterozygosity (He) estimated for the sites under parallel linked selection for each population removing the transitions, the figure is the same as Figure S12a but with all the data points shown. Source data are provided in the Source Data file.

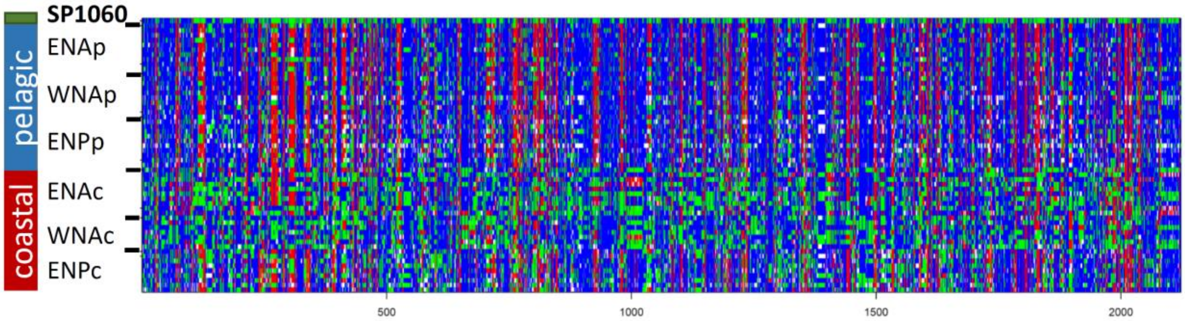


Supplementary Figure 13. Genotypes of the SNPs under repeated selection to coastal habitat in contemporary individuals and ancient individual SP1060. These SNPs include 2,122 SNPs with no missing data in SP1060 out of the 7,165 SNPs identified in Louis *et al.* (2021). Plot of the homozygote reference genotypes in blue, heterozygote in green and homozygote derived in red. Populations include coastal and pelagic ecotypes from the eastern North Atlantic (ENAc and ENAp), western North Atlantic (WNAc and WNAp) and eastern North Pacific (ENPc and ENPp). Source data are provided in the DataSuds repository.

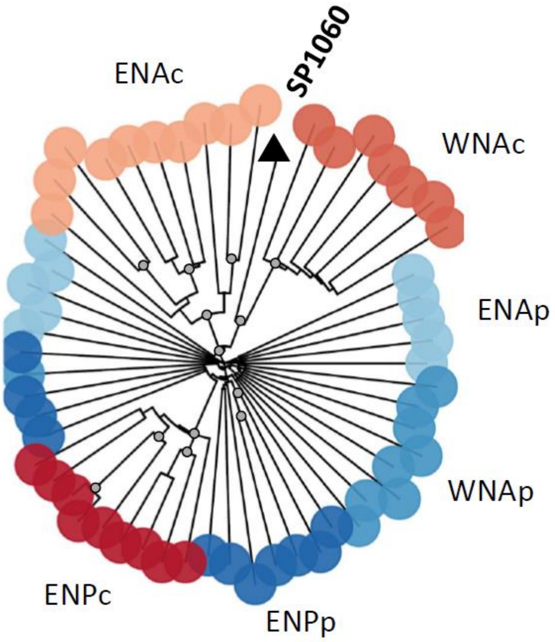


Supplementary Figure 14. Patterns of genetic variation of 2,122 neutral SNPs in contemporary individuals and ancient individual SP1060 (black triangle). Neighbour-joining distance tree showing the genetic structure of the common bottlenose dolphin samples for this particular SNP set, which show no missing data in SP1060. Grey circles indicate bootstrap node support values higher than 95%. Populations include coastal and pelagic ecotypes from the eastern North Atlantic (ENAc and ENAp), western North Atlantic (WNAc and WNAp) and eastern North Pacific (ENPc and ENPp). Source data are provided in the DataSuds repository.

# Supplementary Tables

Supplementary Table 1. a) Ancient subfossil specimen collection number, site location, radio-carbon dating reference code, radiocarbon-dating age (years BP), recalibrated age (years BP) for 1 sigma (1σ, 68% CI) and 2 sigma (2σ, 95% CI). b) Modern sample identification number, collection site, date, type of sample and sex. Biosample (SAMNxxx) and SRA (SRRxxx) accession numbers (AN) to access the raw sequencing data.

a)

| **Sample** | **Site** | **Code** | **$^{14}$C Age (BP)** | **Calibrated $^{14}$C age (BP) [1σ]** | **Calibrated $^{14}$C age (BP) [2σ]** | **AN** |
|---|---|---|---|---|---|---|
| **SP1060** | Southern Bight | R-EVA 1656 | 5,624 +/- 28 | 5,896-5,723 | 5,979-5,626 | SAMN34041706<br>SRR24189295<br>SRR24189284<br>SRR24189275<br>SRR24189296 |
| **NMR10326** | Smiths Knoll | GrA 25851 | 8,135 +/- 45 | 8,518-8,346 | 8,610-8,243 | SAMN34041710<br>SRR24189287_94<br>SRR24189269 |
| **NMR2273** | Smiths Knoll | GrA 25850 | 7,390 +/- 50 | 7,745 -7,572 | 7,842-7,492 | SAMN34041708<br>SRR24189270_74 |
| **NMR10151** | Southern Bight, 52:53:842N 2:83:471E | OxA-32156 | 6,822 +/- 39 | 7,228-7,036 | 7,303-6,935 | SAMN34041707<br>SRR24189279_83<br>SRR24189285_86 |

b)

| **Sample** | **Site** | **Date** | **Type of sample** | **Sex** | **AN** |
|---|---|---|---|---|---|
| **tt0702** | Shannon Estuary, Ireland | 16-07-2007 | biopsy | M | SAMN34041800<br>SRR24189278 |
| **C60** | Bay of Mont St Michel, France | 29-07-2011 | biopsy | F | SAMN34041801<br>SRR24189277 |
| **SW1997/171b** | South Uist, Scotland, United-Kingdom | 20-11-1997 | stranded dolphin | M | SAMN34041802<br>SRR24189276<br>SRR24866568 |

Supplementary Table 2. Summary statistics for the nuclear data for a) the ancient samples and b) the three new contemporary samples. "Ref" indicates the reference genome used for mapping (Tt for *T. truncatus* and Oo for *O. orca*), "sample" indicate sample name, "nb" the number of libraries prepared for that samples, "total reads" the raw number of reads, "mapped N" the number of reads mapped to the nuclear reference genome, "mapped N q25 unique" the number of reads mapped to the nuclear reference genome after mapping quality filter of 25 and removing duplicates, "%N" is the endogenous content after mapping quality and duplicate filters, "effective coverage" represents the coverage after all filters (i.e. post-QC filtering, repeat masking, removing duplicates, base quality recalibration, regions of excessive coverage, sex chromosomes and scaffolds shorter than 10 Mbp, see details in Louis *et al.* (2021) [1]).

a)

| **reference** | **sample** | **nb** | **total reads** | **mapped N** | **mapped N q25 unique** | **% N** | **effective coverage** |
|---|---|---|---|---|---|---|---|
| *T. truncatus* | SP1060 | 4 | 527,270,096 | 225,493,587 | 147,583,308 | 27.99 | 2.841 |
| *O. orca* | SP1060 | 4 | 527,270,096 | 191,133,611 | 127,139,634 | 24.11 | 2.976 |
| *T. truncatus* | NMR2273 | 5 | 87,203,486 | 57,760,143 | 744,073 | 0.85 | 0.008 |
| *O. orca* | NMR2273 | 5 | 87,203,486 | 46,032,421 | 544,427 | 0.62 | 0.001 |
| *T. truncatus* | NMR10326 | 9 | 113,642,049 | 97,736,589 | 1,658,557 | 1.46 | 0.023 |
| *O. orca* | NMR10326 | 9 | 113,642,049 | 79,607,214 | 1,254,302 | 1.10 | 0.002 |
| *T. truncatus* | NMR10151 | 7 | 83,440,588 | 50,960,537 | 102,666 | 0.12 | 0.001 |
| *O. orca* | NMR10151 | 7 | 83,440,588 | 43,138,459 | 99,870 | 0.12 | 0.001 |

b)

| **reference** | **sample** | **nb** | **total reads** | **mapped N q25 unique** | **effective coverage** |
|---|---|---|---|---|---|
| ***T. truncatus*** | Normandy | 1 | 75,898,164 | 59,925,176 | 1.732 |
| ***O. orca*** | Normandy | 1 | 75,898,164 | 59,892,951 | 1.745 |
| ***T. truncatus*** | West Scotland | 1 | 156,592,102 | 119,308,303 | 3.432 |
| ***O. orca*** | West Scotland | 1 | 156,592,102 | 118,514,741 | 3.439 |
| ***T. truncatus*** | Shannon | 1 | 97,573,706 | 74,098,872 | 2.912 |
| ***O. orca*** | Shannon | 1 | 97,573,706 | 73,958,864 | 2.218 |

Supplementary Table 3. Whole mtDNA sequences/mitochondrial haplotypes downloaded from GenBank and used in the phylogenetic and coalescent analyses. Only the protein coding regions from these sequences were used in the estimation of time-calibrated phylogenies for delphinids and for *T. truncatus*.

| **Delphinid phylogeny** | | |
|---|---|---|
| **Species** | **Accession number** | **GenBank reference** |
| *Cephalorhynchus heavisidii* | JN632624 | Hassanin *et al.*, 2012 [5] |
| *Orcaella brevirostris* | JF289177 | Vilstrup *et al.*, 2011 [6] |
| *Orcaella heinsohni* | JF339977 | Vilstrup *et al.*, 2011 |
| *Peponocephala electra* | JF289175 | Vilstrup *et al.*, 2011 |
| *Feresa attenuata* | JF289171 | Vilstrup *et al.*, 2011 |
| *Globicephala melas* | JF339972 | Vilstrup *et al.*, 2011 |
| *Globicephala macrorhynchus* | JF339976 | Vilstrup *et al.*, 2011 |
| *Pseudorca crassidens* | JF289173 | Vilstrup *et al.*, 2011 |
| *Grampus griseus* | EU557095 | Xiong *et al.*, 2009 [7] |
| *Stenella attenuata* | EU557096 | Xiong *et al.*, 2009 |
| *Stenella coeruleoalba* | EU557097 | Xiong *et al.*, 2009 |
| *Delphinus capensis* | EU557094 | Xiong *et al.*, 2009 |
| *Sousa chinensis* | EU557091 | Xiong *et al.*, 2009 |
| *Lagenorhynchus albirostris* | NC005278 | Arnason *et al.*, 2004 [8] |
| *Orcinus orca*, resident ecotype | GU187192 | Morin *et al.*, 2010 [9] |
| *Orcinus orca*, transient ecotype | GU187173 | Morin *et al.*, 2010 |
| *Steno bredanensis* | JF339982 | Vilstrup *et al.*, 2011 |
| *Tursiops aduncus* | KF570335 | Moura *et al.*, 2013 [4] |
| *Tursiops australis* | KF570363 | Moura *et al.*, 2013 |

| ***Tursiops truncatus* phylogeny** | | | |
|---|---|---|---|
| **Species** | **Haplotype name** | **Accession number** | **GenBank reference** |
| *T. truncatus* | EMED3 | KF570315 | Moura *et al.*, 2013 |
| *T. truncatus* | EMED4 | KF570316 | Moura *et al.*, 2013 |
| *T. truncatus* | EMED5 | KF570317 | Moura *et al.*, 2013 |
| *T. truncatus* | EMED1 | KF570318 | Moura *et al.*, 2013 |
| *T. truncatus* | EMED2 | KF570319 | Moura *et al.*, 2013 |
| *T. truncatus* | EMED10 | KF570320 | Moura *et al.*, 2013 |
| *T. truncatus* | EMED6 | KF570321 | Moura *et al.*, 2013 |
| *T. truncatus* | EMED9 | KF570322 | Moura *et al.*, 2013 |
| *T. truncatus* | EMED7 | KF570323 | Moura *et al.*, 2013 |
| *T. truncatus* | EMED8 | KF570324 | Moura *et al.*, 2013 |
| *T. truncatus ponticus* | BSEA2 | KF570325 | Moura *et al.*, 2013 |
| *T. truncatus ponticus* | BSEA3 | KF570326 | Moura *et al.*, 2013 |
| *T. truncatus ponticus* | BSEA1 | KF570327 | Moura *et al.*, 2013 |
| *T. truncatus ponticus* | BSEA6 | KF570328 | Moura *et al.*, 2013 |
| *T. truncatus ponticus* | BSEA7 | KF570329 | Moura *et al.*, 2013 |
| *T. truncatus ponticus* | BSEA5 | KF570330 | Moura *et al.*, 2013 |
| *T. truncatus ponticus* | BSEA4 | KF570331 | Moura *et al.*, 2013 |
| *T. truncatus ponticus* | BSEA8 | KF570332 | Moura *et al.*, 2013 |
| *T. truncatus ponticus* | BSEA9 | KF570333 | Moura *et al.*, 2013 |
| *T. truncatus ponticus* | BSEA10 | KF570334 | Moura *et al.*, 2013 |
| *T. truncatus* | SCO7 | KF570345 | Moura *et al.*, 2013 |
| *T. truncatus* | SCO1 | KF570346 | Moura *et al.*, 2013 |
| *T. truncatus* | SCO6 | KF570347 | Moura *et al.*, 2013 |
| *T. truncatus* | SCO2 | KF570348 | Moura *et al.*, 2013 |
| *T. truncatus* | SCO3 | KF570349 | Moura *et al.*, 2013 |

| | | | |
|---|---|---|---|
| *T. truncatus* | SCO4 | KF570350 | Moura *et al.*, 2013 |
| *T. truncatus* | SCO8 | KF570351 | Moura *et al.*, 2013 |
| *T. truncatus* | SCO5 | KF570352 | Moura *et al.*, 2013 |
| *T. truncatus* | CG1 | KF570389 | Moura *et al.*, 2013 |
| *T. truncatus* | WNAP9 | KF570388 | Moura *et al.*, 2013 |
| *T. truncatus* | WNAP8 | KF570387 | Moura *et al.*, 2013 |
| *T. truncatus* | WNAP7 | KF570386 | Moura *et al.*, 2013 |
| *T. truncatus* | WNAP26 | KF570385 | Moura *et al.*, 2013 |
| *T. truncatus* | WNAP22 | KF570384 | Moura *et al.*, 2013 |
| *T. truncatus* | WNAP21 | KF570383 | Moura *et al.*, 2013 |
| *T. truncatus* | WNAP19 | KF570382 | Moura *et al.*, 2013 |
| *T. truncatus* | WNAP17 | KF570381 | Moura *et al.*, 2013 |
| *T. truncatus* | WNAP12 | KF570380 | Moura *et al.*, 2013 |
| *T. truncatus* | WNAP11 | KF570379 | Moura *et al.*, 2013 |
| *T. truncatus* | WNAC8 | KF570378 | Moura *et al.*, 2013 |
| *T. truncatus* | WNAC25 | KF570377 | Moura *et al.*, 2013 |
| *T. truncatus* | WNAC23 | KF570376 | Moura *et al.*, 2013 |
| *T. truncatus* | WNAC22 | KF570375 | Moura *et al.*, 2013 |
| *T. truncatus* | WNAC19 | KF570374 | Moura *et al.*, 2013 |
| *T. truncatus* | WNAC16 | KF570373 | Moura *et al.*, 2013 |
| *T. truncatus* | WNAC14 | KF570372 | Moura *et al.*, 2013 |
| *T. truncatus* | WNAC13 | KF570371 | Moura *et al.*, 2013 |
| *T. truncatus* | WNAC11 | KF570370 | Moura *et al.*, 2013 |
| *T. truncatus* | ENAC1 (N=5)† | KT601188 | Nykänen *et al.*, 2019 |
| *T. truncatus* | ENAC2 | KT601189 | Nykänen *et al.*, 2019 |
| *T. truncatus* | ENAC3 | KT601190 | Nykänen *et al.*, 2019 |
| *T. truncatus* | ENAC4 | KT601191 | Nykänen *et al.*, 2019 |

| | | | |
|---|---|---|---|
| *T. truncatus* | ENAC5 | KT601192 | Nykänen *et al*., 2019 |
| *T. truncatus* | ENAC6 (N=2)† | KT601193 | Nykänen *et al*., 2019 |
| *T. truncatus* | ENAC7 | KT601194 | Nykänen *et al*., 2019 |
| *T. truncatus* | ENAC8 | KT601195 | Nykänen *et al*., 2019 |
| *T. truncatus* | ENAC9 | KT601196 | Nykänen *et al*., 2019 |
| *T. truncatus* | ENAP1 | KT601197 | Nykänen *et al*., 2019 |
| *T. truncatus* | ENAP2 | KT601198 | Nykänen *et al*., 2019 |
| *T. truncatus* | ENAP3 | KT601199 | Nykänen *et al*., 2019 |
| *T. truncatus* | ENAP4 (N=6)† | KT601200 | Nykänen *et al*., 2019 |
| *T. truncatus* | ENAP5 | KT601201 | Nykänen *et al*., 2019 |
| *T. truncatus* | ENAP6 | KT601202 | Nykänen *et al*., 2019 |
| *T. truncatus* | ENAP7 | KT601203 | Nykänen *et al*., 2019 |
| *T. truncatus* | ENAP8 | KT601204 | Nykänen *et al*., 2019 |
| *T. truncatus* | ENAP9 | KT601205 | Nykänen *et al*., 2019 |
| *T. truncatus* | ENAP10 | KT601206 | Nykänen *et al*., 2019 |
| *T. truncatus* | ENAP11 | KT601207 | Nykänen *et al*., 2019 |

† GenBank haplotype plus samples from Nykänen *et al.* (2019) that were duplicates to the haplotype

Supplementary Table 4. Best partitioning schemes for nucleotide substitution models used in the construction of the delphinid and *T. truncatus* time-calibrated phylogenies.

| **Delphinids: All codon model** | | |
|---|---|---|
| **Partition** | **Best model** | **Subset partitions** |
| p1 | HKY+I+G | atp6, atp8, cox2, cox3, cytb, nd1, nd2, nd3, nd4, nd4l, nd5 |
| p2 | HKY+I+G | cox1 |
| p3 | HKY+I+G | nd6 |
| | | |
| Delphinids: Third codon only model | | |
| **Partition** | **Best model** | **Subset partitions** |
| p1 | TrN+I+G | atp6, atp8, cox3, cytb, nd1, nd2, nd3, nd4, nd4l, nd5 |
| p2 | TrN+G | atp8, cox1 |
| p3 | TrN+G | nd6 |
| | | |
| *T. truncatus*: All codon model | | |
| **Partition** | **Best model** | **Subset partitions** |
| p1 | HKY+I | atp6, atp8, cox3, cytb, nd1, nd2, nd3, nd4, nd4l, nd5 |
| p2 | HKY+G | cox1, cox2 |
| p3 | HKY+I | nd6 |
| | | |
| *T. truncatus*: Third codon only model | | |
| **Partition** | **Best model** | **Subset partitions** |
| p1 | TrN | cox3, cytb, nd1, nd2, nd4, nd4l, nd5 |
| p2 | TrN | atp6, atp8, cox1, cox2, nd3 |
| p3 | TrN+I | nd6 |

Supplementary Table 5. Different coalescent scenarios run with BEAST2 to estimate *T. truncatus* phylogeny.

| **Tree scenario** | **Model** | **Root calibration, mean (SD)** | **Tip calibration, mean (SD)** | | |
|---|---|---|---|---|---|
| | | | **2273** | **3920** | **10151** |
| Without 117696, without 10151 | All codon | 0.9162 (0.158) | 0.007663 (1.00E-04) | 0.00843 (1.00E-04) | NA |
| Without 117696, without 10151 | 3rd codon | 0.7767 (0.145) | 0.007663 (1.00E-04) | 0.00843 (1.00E-04) | NA |
| Without 117696, with 10151 | All codon | 0.9162 (0.158) | 0.007663 (1.00E-04) | 0.00843 (1.00E-04) | 0.007126 (1.00E-04) |
| Without 117696, with 10151 | 3rd codon | 0.7767 (0.145) | 0.007663 (1.00E-04) | 0.00843 (1.00E-04) | 0.007126 (1.00E-04) |
| With 117696, without 10151 | All codon | 1.174 (0.2) | 0.007663 (1.00E-04) | 0.00843 (1.00E-04) | NA |
| With 117696, without 10151 | 3rd codon | 1.0332 (0.18) | 0.007663 (1.00E-04) | 0.00843 (1.00E-04) | NA |
| With 117696 and 10151 | All codon | 1.174 (0.2) | 0.007663 (1.00E-04) | 0.00843 (1.00E-04) | 0.007126 (1.00E-04) |
| With 117696 and 10151 | 3rd codon | 1.0332 (0.18) | 0.007663 (1.00E-04) | 0.00843 (1.00E-04) | 0.007126 (1.00E-04) |

## Supplementary References

1. Louis, M. *et al.* Selection on ancestral genetic variation fuels repeated ecotype formation in bottlenose dolphins. *Sci Adv* **7**, eabg1245 (2021).

2. Costa, A. P. B., Mcfee, W. & Wilcox, L. A. The common bottlenose dolphin (*Tursiops truncatus*) ecotypes of the western North Atlantic revisited: an integrative taxonomic investigation supports the presence of distinct species. *Zool. J. Linn. Soc.* (2022) doi:10.1093/zoolinnean/zlac025/6585199.

3. Nykänen, M. *et al.* Postglacial colonization of northern coastal habitat by bottlenose dolphins: a marine leading-edge expansion? *J. Hered.* **110**, 662–674 (2019).

4. Moura, A. E. *et al.* Recent diversification of a marine genus (*Tursiops spp.*) tracks habitat preference and environmental change. *Syst. Biol.* **62**, 865–877 (2013).
5. Hassanin, A. *et al.* Pattern and timing of diversification of Cetartiodactyla (Mammalia, Laurasiatheria), as revealed by a comprehensive analysis of mitochondrial genomes. *C. R. Biol.* **335**, 32–50 (2012).
6. Vilstrup, J. T. *et al.* Mitogenomic phylogenetic analyses of the Delphinidae with an emphasis on the Globicephalinae. *BMC Evol. Biol.* **11**, 65 (2011).
7. Xiong, Y., Brandley, M. C., Xu, S., Zhou, K. & Yang, G. Seven new dolphin mitochondrial genomes and a time-calibrated phylogeny of whales. *BMC Evol. Biol.* **9**, 20 (2009).
8. Arnason, U., Gullberg, A. & Janke, A. Mitogenomic analyses provide new insights into cetacean origin and evolution. *Gene* **333**, 27–34 (2004).
9. Morin, P. A. *et al.* Complete mitochondrial genome phylogeographic analysis of killer whales (Orcinus orca) indicates multiple species. *Genome Res.* **20**, 908–916 (2010).
