# Supplementary material for: Ancient dolphin genomes reveal rapid repeated adaptation to coastal waters
Source: Nat Commun. 2023 Jul 18;14:4020. doi: 10.1038/s41467-023-39532-z (PMC10354069; doi:10.1038/s41467-023-39532-z)
Supplement: Supplementary file 3 — Description of Additional Supplementary Files [file 41467_2023_39532_MOESM3_ESM.pdf]

Ancient dolphin genomes reveal rapid repeated adaptation to coastal waters

Description of Additional Supplementary Materials

Supplementary Data 1

It include three data sheets

The first sheet “nuclear_ancient” include all the information we have on the ancient specimens, also included in Table S1 as well as where the specimens are hosted, the contact person and the link to the raw sequencing data.

The second sheet “nuclear_contemporary” include all the information we have on the contemporary specimens, also included in Table S1 as well as where the specimens are hosted, the contact person and the link to the raw sequencing data.

The third sheet “mitogenomes” include the list of individuals and their mitogenome sequences ID and accession number.

DataSuds repository: data and code availability

The information below is also available in the Readme file on the DataSuds repository https://doi.org/10.23708/DABAWD

See methods section for details.

File “code_mapping_filtering_ancientDNA.txt” provides the pipeline to map and filter the ancient DNA data

File “code_mapping_filtering_contemporaryDNA.txt” provides the pipelone to map and filter the contemporary DNA data, including the three new samples, and the outgroups (killer whale and Indo-Pacific bottlenose dolphin)

File “code_data_analyses.txt” provides the scripts and data to reproduce all the analyses in the manuscript

The folders are linked to the file “code_data_analyses.txt” and provide the files required to generate the analyses, most scripts are in “code_data_analyses.txt”

Folder “map” provides the R script and files to plot the samples (Figure 1a)

Folder “3.1.1.smartpca_fromvcf” provides the file to format a vcf file and run smartpca (Figure S4)

Folder “3.1.2.smartpca_fromhaplo” provides the file to format an haplo file and run smartpca both for the data mapped to the killer whale reference genome and bottlenose dolphin reference genome (see subfolder “mapped_Ttruncatus”) (Figures 1c, S5 and S6)

Folder “3.2.single_read_PCA” provides the file to run and plot a single-read PCA (Figure S8)

Folder “3.3.tfa” provides the file to run and plot a factor analysis (Figures 1d and S7)

Folder “3.4.PCA_downsampling_modern” provides the files to run smartpca (subfolder “3.4.1.smartpca”) and the single read PCA (subfolder “3.4.2.single_read”) on the modern data including one individual downsampled to 0.03x (Figure S3)

Folder “4.1.D_statistics” provides the files to run D-statistics (Figures S9 and S10)

Folder “4.2.qpbrute” provides the file to format the files and run qpbrute from an haplo file (subfolder “4.2.1.pseudohaploid_withSP1060”) or a vcf file (“subfolder 4.2.2.genotypes_modern”) (Figure 2 and S11)

Folder “5.selection” provides the files to run and plot heterozygosity (subfolder “5.1.heterozygosity”), and genotype plots, NJ tree (including bootstrapping) and PCA (subfolder “P5.2.CA_NJtree_genotype_plot”) for the SNPs under parallel linked selection. There is also a subfolder within 5.2. to generate a NJ tree for the same number of randomly sampled neutral SNPs. This produces Figures 3, S12, S13 and S14.

Folder 6.mitogenomes provides the files to generate the mitogenone analyses (Figure S1)
