# Supplementary material for: Ancient dolphin genomes reveal rapid repeated adaptation to coastal waters
Source: Nat Commun. 2023 Jul 18;14:4020. doi: 10.1038/s41467-023-39532-z (PMC10354069; doi:10.1038/s41467-023-39532-z)
Supplement: Supplementary file 5 — Reporting Summary [file 41467_2023_39532_MOESM5_ESM.pdf]

nature portfolio

Corresponding author(s): Marie Louis, Andrew D. Foote

Last updated by author(s): Jun 13, 2023

Reporting Summary

Nature Portfolio wishes to improve the reproducibility of the work that we publish. This form provides structure for consistency and transparency in reporting. For further information on Nature Portfolio policies, see our Editorial Policies and the Editorial Policy Checklist.

Statistics

For all statistical analyses, confirm that the following items are present in the figure legend, table legend, main text, or Methods section.

n/a	Confirmed	
☐	☒	The exact sample size (*n*) for each experimental group/condition, given as a discrete number and unit of measurement
☐	☒	A statement on whether measurements were taken from distinct samples or whether the same sample was measured repeatedly
☐	☒	The statistical test(s) used AND whether they are one- or two-sided<br>*Only common tests should be described solely by name; describe more complex techniques in the Methods section.*
☒	☐	A description of all covariates tested
☐	☒	A description of any assumptions or corrections, such as tests of normality and adjustment for multiple comparisons
☐	☒	A full description of the statistical parameters including central tendency (e.g. means) or other basic estimates (e.g. regression coefficient) AND variation (e.g. standard deviation) or associated estimates of uncertainty (e.g. confidence intervals)
☐	☒	For null hypothesis testing, the test statistic (e.g. *F*, *t*, *r*) with confidence intervals, effect sizes, degrees of freedom and *P* value noted<br>*Give P values as exact values whenever suitable.*
☐	☒	For Bayesian analysis, information on the choice of priors and Markov chain Monte Carlo settings
☒	☐	For hierarchical and complex designs, identification of the appropriate level for tests and full reporting of outcomes
☒	☐	Estimates of effect sizes (e.g. Cohen's *d*, Pearson's *r*), indicating how they were calculated

Our web collection on statistics for biologists contains articles on many of the points above.

Software and code

Policy information about availability of computer code

Data collection	We did not collect data with softwares.
Data analysis	We have used the following softwares:<br>CALIB v.8.2<br>Illumina's CASAVA v.1.8.2<br>ADAPTER-REMOVAL v.2<br>Trimmomatic v.0.32<br>BWA v.0.7.15<br>samtools v.1.2<br>ANGSD v.0.913<br>ANGSD v.0.921<br>picard-tools v.2.1.0<br>MEGA X<br>bedtools v.2.25.0<br>MapDamage v.2.0<br>MrBayes v.3.2.7a<br>jModelTest2 v2.1.10<br>PartitionFinder v.1.1.0<br>BEAST2 v.2.6

March 2021

Tracer v.1.7.1
TreeAnnotator v.2.6
LogCombiner v.2.6
R v.4.0.5
R package CODA v.0.19-4
R package adegenet v.2.1.5
R package scales v.1.2.1
R package vcfR v.1.12.0
R package ape v.5.0 and v.5.6.2
R package LEA v.3.2.0
R package tfa
FigTree v.1.44
Eigensoft v.6.1.3
Eigensoft v.7.2.0
https://github.com/mathii/gdc/blob/master/vcf2eigenstrat.py
Plink v.1.9
qpBrute (https://github.com/ekirving/qpbrute)
R package ADMIXTUREGRAPH v.1.0.2
Input files and scripts are available on the DataSuds repository: https://doi.org/10.23708/DABAWD.

For manuscripts utilizing custom algorithms or software that are central to the research but not yet described in published literature, software must be made available to editors and reviewers. We strongly encourage code deposition in a community repository (e.g. GitHub). See the Nature Portfolio guidelines for submitting code & software for further information.

Data

Policy information about availability of data

All manuscripts must include a data availability statement. This statement should provide the following information, where applicable:

- Accession codes, unique identifiers, or web links for publicly available datasets
- A description of any restrictions on data availability
- For clinical datasets or third party data, please ensure that the statement adheres to our policy

Raw sequencing data generated as part of this project are part of Bioproject PRJNA955223: https://www.ncbi.nlm.nih.gov/bioproject/PRJNA955223. Biosample and SRA accession numbers are provided in Supplementary Table 1 and Supplementary data file 1. Mitochondrial genome sequences are accessible on GenBank at accession numbers OR120165-OR120228 as detailed in Supplementary data file 1. Supplementary data file 1 also indicates where the samples are housed and who should be contacted to access the material. We also used the whole-genome re-sequencing data from Bioproject PRJNA724031. We have also included mitogenome sequences from Genbank as indicated in Supplementary Table 3. Source data are provided with this paper in the Source Data file and the data, codes and related documentations that support the findings of this study are openly available in DataSuds repository (IRD, France) at https://doi.org/10.23708/DABAWD. Data reuse is granted under CC-BY licence.

Human research participants

Policy information about studies involving human research participants and Sex and Gender in Research.

Reporting on sex and gender	not applicable
Population characteristics	*Describe the covariate-relevant population characteristics of the human research participants (e.g. age, genotypic information, past and current diagnosis and treatment categories). If you filled out the behavioural & social sciences study design questions and have nothing to add here, write "See above."*
Recruitment	*Describe how participants were recruited. Outline any potential self-selection bias or other biases that may be present and how these are likely to impact results.*
Ethics oversight	*Identify the organization(s) that approved the study protocol.*

Note that full information on the approval of the study protocol must also be provided in the manuscript.

Field-specific reporting

Please select the one below that is the best fit for your research. If you are not sure, read the appropriate sections before making your selection.

☐ Life sciences ☐ Behavioural & social sciences ☒ Ecological, evolutionary & environmental sciences

For a reference copy of the document with all sections, see nature.com/documents/nr-reporting-summary-flat.pdf

Ecological, evolutionary & environmental sciences study design

All studies must disclose on these points even when the disclosure is negative.

Study description	We investigate the chronology and independence of adaptation to coastal habitat from standing genetic variation in common bottlenose dolphins derived from pelagic ancestors using 60 contemporary and four ancient (8,610-5,626 years before present (BP)) genomes.
Research sample	Our samples include whole genome re-sequencing data from four bottlenose dolphin subfossil specimens dated to 8,610-5,626 years before present, please see Table S1 for details about each specimen, and three contemporary bottlenose dolphins from neighboring coastal populations in the eastern North Atlantic (Shannon Estuary, Ireland, Bay of Mont St Michel, France, and South Uist, Scotland, United-Kingdom). We sequenced the subfossil specimens based on endogenous content and radiocarbon dating information availability. We did not know a priori to which population the samples belong. We also include already published whole genome resequencing data from 57 coastal and pelagic bottlenose dolphins from the eastern North Atlantic, western North Atlantic and eastern North Pacific from Louis et al., 2021 in Science Advances. For the mitogenome analyses all those samples are included, as well as published data from Nykänen et al. 2019 Journal of Heredity and Moura et al. 2013 Systematic Biology for the bottlenose dolphin phylogeny and from Hassanin et al., 2012 C. R. Biol.; Vilstrup et al., 2011 BMC Evol. Biol; Xiong et al., 2009 BMC Evol. Biol; Arnason et al., 2004 Gene and Morin et al., 2010 Genome Res for the delphinid phylogeny (see details in Table S3).
Sampling strategy	We used 7-10 samples per ecotype per region in Louis et al. 2021 as such sample size is considered sufficient to run population genomic analyses based on allele frequencies from whole genome re-sequencing data and to run analyses using mitochondrial genomes. We have used 1 to 4 ancient samples in our analyses, and used methods which are appropriate for low sample size. Low sample size is common in ancient DNA analyses due to the degradation of the material which makes it difficult to recover DNA from all specimens. We sequenced the subfossil specimens based on endogenous content and radiocarbon dating information availability. We randomly chose one individual for each of the eastern North Atlantic coastal populations.
Data collection	We collected bone powder from subfossil bottlenose dolphin specimens curated at the Natural History Museum of Rotterdam (Table S1). We collected skin samples from contemporary bottlenose dolphins by biopsy sampling or sampling a stranded dolphin already dead on the coast.
Timing and spatial scale	The subfossil samples were sampled at the Natural History Museum of Rotterdam and aged between 8,610-5,626 years before present (Table S1). The subfossil samples were dredged from the southern part of the North Sea by commercial trawlers. The three new modern samples were collected between 1997 and 2011 in Scotland, France and Ireland in the eastern North Atlantic (Table S1). Samples from Louis et al., 2021 in Science Advances include 57 coastal and pelagic bottlenose dolphins collected between 1992 and 2015 in the eastern North Atlantic, western North Atlantic and eastern North Pacific.<br>Bottlenose dolphins have long generation time and low reproduction rate and therefore sampling over two decades does not represent an issue for our study.
Data exclusions	We did not exclude any data. However, some analyses include all ancient samples and others only the ancient sample with the highest coverage (SP1060), depending on coverage requirement for the analyses.
Reproducibility	Our study did not include any experiment. We provide all the scripts to reproduce the results of the study.
Randomization	Randomization is not applicable for most of our analyses. We assigned samples to ecotype in each region based on morphology or previous genetic analyses using microsatellite markers. We confirmed ecotype/population assignment using PCA and admixture analyses. We performed bootstrapping for one of our analyses (the neighbour-joining tree Figure 3).
Blinding	Investigators were not blinded to the origin of the samples. However, data were analysed with no a priori based on sample origin. The ecotype assignment of the ancient dolphins was unknown prior to genomic analyses. We used the same data processing pipeline on all samples, with some modifications specific to ancient DNA data for the subfossil samples (e.g. recalibration of base quality scores based on DNA damage patterns).

Did the study involve field work? ☒ Yes ☐ No

Field work, collection and transport

Field conditions	Bottlenose dolphin samples were collected previously for other studies when the weather conditions were good, that is with no or low wind.
Location	They were collected in coastal waters generally less than 20m in the Shannon Estuary, Ireland, Bay of Mont St Michel, France, and South Uist, Scotland, United-Kingdom.
Access & import/export	The three new contemporary dolphin samples analysed in this study were collected under the relevant permits of each country. Biopsy sampling was carried out under licence from the National Parks and Wildlife Service in Ireland and under licence from the Direction Regionale de l'Environnement, de l'Amenagement et du Logement in France. DNA samples were shipped within Europe (prior to Brexit) and therefore no CITES permits were required.
Disturbance	All biopsy samples were taken under a standard protocol, for which impact studies reported that the behavioural reactions of

dolphins were limited and only short-term, and no healing complications or infections were reported (Weller et al. 1997 Aquat Mamm; Krützen et al. 2002 Mar Mam Sci; Kiszka et al. 2010 Animal Welfare; Tezanos-Pinto & Baker 2011 New Zeal J Mar Fresh).

Reporting for specific materials, systems and methods

We require information from authors about some types of materials, experimental systems and methods used in many studies. Here, indicate whether each material, system or method listed is relevant to your study. If you are not sure if a list item applies to your research, read the appropriate section before selecting a response.

Materials & experimental systems

n/a	Involved in the study
☒	☐ Antibodies
☒	☐ Eukaryotic cell lines
☐	☒ Palaeontology and archaeology
☐	☒ Animals and other organisms
☒	☐ Clinical data
☒	☐ Dual use research of concern

Methods

n/a	Involved in the study
☒	☐ ChIP-seq
☒	☐ Flow cytometry
☒	☐ MRI-based neuroimaging

Palaeontology and Archaeology

Specimen provenance: The four common bottlenose dolphin subfossil samples were dredged from the Southern part of the North Sea (i.e., Southern Bight and Smiths Knoll) by commercial trawlers. As they were side products of commercial fishing, no permit was required.

Specimen deposition: The specimens are deposited at the Natural History Museum of Rotterdam.

Dating methods: Radiocarbon dating was performed in previous studies at the Klaus-Tschira-AMS facility for SP1060 (Korlević et al. 2018 Sci. Rep.) and at the University of Groningen for NMR2273 and NMR10326 (Kompanje and Post 2017, Lutra). For NMR10151, we performed radiocarbon dating at the University of Oxford.
We re-calibrated the age of all the samples using the marine20 correction in CALIB 8.2 applying a ΔR (i.e. localised reservoir correction) of 8±38 as estimated for odontocete bones in Norway (Mangerud et al. 2006). We provide this information in the supplementary material and table S1.

☒ Tick this box to confirm that the raw and calibrated dates are available in the paper or in Supplementary Information.

Ethics oversight: We sampled the specimens according to the Natural History Museum of Rotterdam guidelines.

Note that full information on the approval of the study protocol must also be provided in the manuscript.

Animals and other research organisms

Policy information about studies involving animals; ARRIVE guidelines recommended for reporting animal research, and Sex and Gender in Research

Laboratory animals: No laboratory animals were used.

Wild animals: We collected biopsy samples (a small piece of skin and blubber) from three wild bottlenose dolphins under a standard protocol which has limited impact on the animals (Weller et al. 1997 Aquat Mamm; Krützen et al. 2002 Mar Mam Sci; Kiszka et al. 2010 Animal Welfare; Tezanos-Pinto & Baker 2011 New Zeal J Mar Fresh).

Reporting on sex: Sex information is not relevant for our analyses, sex information is provided for the three new samples for which we generated whole genome resequencing data in Table S1.

Field-collected samples: Skin samples were either frozen or stored in 95% ethanol.

Ethics oversight: The three new contemporary dolphin samples analysed in this study were collected under the relevant permits of each country. Biopsy sampling was carried out under licence from the National Parks and Wildlife Service in Ireland and under licence from the Direction Regionale de l'Environnement, de l'Amenagement et du Logement in France. The sample in West Scotland was collected by the Scottish Marine Animal Stranding Scheme, from a stranded animal which was found dead on the shore, in accordance with the law in the country.

Note that full information on the approval of the study protocol must also be provided in the manuscript.
